# Uremic myopathy: is oxidative stress implicated in muscle dysfunction in uremia?

**DOI:** 10.3389/fphys.2015.00102

**Published:** 2015-03-30

**Authors:** Antonia Kaltsatou, Giorgos K. Sakkas, Konstantina P. Poulianiti, Yiannis Koutedakis, Konstantinos Tepetes, Grigorios Christodoulidis, Ioannis Stefanidis, Christina Karatzaferi

**Affiliations:** ^1^Department of Physical Education and Sport Sciences (DPESS), School of Physical Education (PE), University of ThessalyTrikala, Greece; ^2^Institute for Research and Technology-Centre for Research and Technology HellasTrikala, Greece; ^3^Department of Surgery, Faculty of Medicine, University of ThessalyLarissa, Greece; ^4^Department of Nephrology, Faculty of Medicine, University of ThessalyLarissa, Greece

**Keywords:** oxidative stress, uremia, muscle dysfunction, uremic myopathy, premature fatigue, muscle weakness

## Abstract

Renal failure is accompanied by progressive muscle weakness and premature fatigue, in part linked to hypokinesis and in part to uremic toxicity. These changes are associated with various detrimental biochemical and morphological alterations. All of these pathological parameters are collectively termed uremic myopathy. Various interventions while helpful can't fully remedy the pathological phenotype. Complex mechanisms that stimulate muscle dysfunction in uremia have been proposed, and oxidative stress could be implicated. Skeletal muscles continuously produce reactive oxygen species (ROS) and reactive nitrogen species (RNS) at rest and more so during contraction. The aim of this mini review is to provide an update on recent advances in our understanding of how ROS and RNS generation might contribute to muscle dysfunction in uremia. Thus, a systematic review was conducted searching PubMed and Scopus by using the Cochrane and PRISMA guidelines. While few studies met our criteria their findings are discussed making reference to other available literature data. Oxidative stress can direct muscle cells into a catabolic state and chronic exposure to it leads to wasting. Moreover, redox disturbances can significantly affect force production *per se*. We conclude that oxidative stress can be in part responsible for some aspects of uremic myopathy. Further research is needed to discern clear mechanisms and to help efforts to counteract muscle weakness and exercise intolerance in uremic patients.

## Introduction

Among the clinical entities affecting thousands of patients, chronic kidney disease (CKD) is a silent epidemic expected to influence more than 50% of the Americans born today (Grams et al., 2013) and approximately 40% of the population in Europe (Zoccali et al., 2010). Muscular weakness, muscle wasting, limited endurance, exercise intolerance, and fatigue are components of the functional and morphological abnormalities collectively termed uremic myopathy, which often also includes uremic cardiomyopathy (Campistol, 2002). While the pathogenesis of uremic myopathy is not clear, it is thought that an interplay of uremic toxicity and hypokinesis guide these abnormalities in patients with CKD and especially in end-stage renal disease (ESRD) patients undergoing hemodialysis (HD) therapy. Observations of a significant correlation between glomerular filtration rate (GFR) and exercise tolerance (e.g., Clyne et al., 1987, 1994) led to studies revealing the very low activity levels and poor functional capacity of renal patients (Kouidi et al., 1998; Johansen et al., 2003; Sakkas et al., 2003a). Moreover, various groups turned their attention to exercise and other interventions to remedy or halt muscle deterioration in pre-dialysis (e.g., Clyne et al., 1991) and dialysis patients (e.g., Sakkas et al., 2003b; Johansen et al., 2006). Despite the evident improvements in exercise capacity and muscle morphology (Sakkas et al., 2003b, 2008a), in increasing muscle mass with steroid supplementation (Topp et al., 2003), in improving sleep and overall quality of life (Sakkas et al., 2008c) it appears that interventions so far cannot restore muscle functionality in ESRD patients to the level of age-matched healthy sedentary individuals (Sakkas et al., 2003b, 2008a; Giannaki et al., 2011).

## Components of uremic myopathy

Loss of skeletal muscle strength in renal patients, contributes to easy fatigability, and can be linked to loss of muscle fibers and atrophy of the remaining fibers (Porter et al., 1995; Sakkas et al., 2003a). In cross-sectional studies, comparing age-matched controls and end-stage patients, atrophy and loss of type IIα and IIx fibers, reduced muscle fiber capillarization and peripheral activation (Sakkas et al., 2003a), and a significant decrease in the mean diameter of both fiber types (Crowe et al., 2007) has been observed. However, not all functional consequences can be attributed to atrophy. Interventions to improve muscle mass indicate that there is a functional deficit in the existing muscle mass. Dialysis patients present with rapid and large accumulation of inorganic phosphate during submaximal exercise, lower oxidative potential, larger phosphocreatine reduction with slower recovery but also with evidence of central activation failure, all these factors contributing to early and excess fatigue (Johansen et al., 2005). Abnormal mitochondria respiratory capacity, is also a factor responsible for easily fatigability in CKD patients, as mitochondrial morphology is disturbed in patients with CKD (Kouidi et al., 1998), while alterations in respiratory chain proteins likely enhance reactive oxygen species (ROS) production which has been seen in a rat uremia model (Yazdi et al., 2013).

CKD patients, especially the end-stage ones, lead a very sedentary lifestyle. Morphological abnormalities however have been observed in both locomotory and non-locomotory muscles (Sakkas et al., 2003a) thus not all of the dysfunction can be attributed to inactivity.

Biochemical and nutritional changes occurring through the progression of CKD can stimulate protein losses and can contribute to the development of muscle wasting. This has grave significance as catabolic conditions increase the risk of morbidity and mortality (Griffiths, 1996; Gordon et al., 2007).

Pro-dialysis and dialysis patients face increasing dietary restrictions. Malnutrition is associated with hypoalbuminemia, which is inversely correlated with mortality in uremic patients (Lowrie and Lew, 1990), and it is also used as a marker of depleted protein stores (Carrero et al., 2008).

Metabolic acidosis, which is commonly associated with CKD, stimulates the breakdown of muscle proteins resulting in loss of muscle mass (Hu et al., 2013). Furthermore, the observation that insulin resistance, is common in patients with CKD, suggests that impaired insulin signaling could also contribute to protein losses (Sakkas et al., 2008b; Zhang et al., 2009). Moreover, CKD is associated with an increase in circulating levels of inflammatory cytokines. Specifically, levels of circulating IL-6, TNF-α, serum amyloid A, and C-reactive protein are increased in patients with CKD (Zhang et al., 2009, 2011; Cheung et al., 2010). Notably, it is contested that in well-dialyzed patients, circulating proinflammatory markers are the main cause for hypoalbuminemia rather than malnutrition (Kaysen et al., 2004). The possibility of an accelerated protein degradation in CKD mediated by the ubiquitin-proteasome system (UPS) (Wang and Mitch, 2013) should also be considered.

Apart from a compounded or accelerated muscle loss, a reduction in the ability to anabolize muscle could be an issue in CKD. Still, interventions with nandrolone decanoate were successful in increasing muscle mass, albeit without improving muscle strength (Topp et al., 2003), pointing to an available anabolic response. However, there are suggestions that CKD may dampen the function of satellite cells. Zhang et al. (2010) using a mouse model of CKD reported a delayed regeneration of damaged muscle and reductions in MyoD protein and the myogenin expression, indicating a decreased satellite cell proliferation and differentiation (Zhang et al., 2010).

To compound the above, in dialyzed patients, the HD procedure *per se* stimulates protein degradation and reduced protein synthesis with the effect persisting for 2 h following dialysis (Ikizler et al., 2002). Thus, while blunting of anabolic responses can't be excluded, a multitude of factors can promote protein loss, especially in the end-stage patients.

## Is there a role for oxidative stress in uremic muscle dysfunction?

Oxidative stress promotes catabolic state and accelerates muscle atrophy (Moylan and Reid, 2007). But it can also affect contractility of the available muscle and sarcomeric protein expression.

Many studies have found that oxidative stress can cause long-term effects and acute effects (Lamb and Westerblad, 2011) on contractility. Long-term effects include altered gene and protein expression or damages in lipids and proteins that are irreversible, while acute effects are reversible. The decrease in Ca^2+^ sensitivity which contributes to muscle fatigue is considered as an acute effect of oxidative stress (Lamb and Westerblad, 2011).

A key mechanism that has been proposed to explain the ROS contribution in muscle fatigue is the reduced myofibrillar Ca^2+^ sensitivity and/or sarcoplasmic reticulum Ca^2+^ release (Allen et al., 2008). Moreover, an increase in NO during fatigue in fast twitch muscle fibers contribute in decreased myofibrillar Ca^2+^ sensitivity (Lamb and Westerblad, 2011). However, in slow-twitch fibers NO donors, did not affect myofibrillar Ca^2+^ sensitivity (Spencer and Posterino, 2009). Also, a study by Reardon and Allen (2009), showed that iron can increase ROS production at high temperature in the skeletal muscle cells, accelerating muscle fatigue.

Moreover, ROS generation can acutely affect contractile function and disturbs structural transition within the actomyosin complex which is crucial for force generation. Exposure to low or high concentrations of peroxide (5 or 50 mM) reduces maximum force and velocity of contraction, with the high peroxide resulting in irreversible loss of calcium regulation of force mediated by oxidation of methionines in the heavy and essential light chains (Prochniewicz et al., 2008a). More elegant work from same group, examining structural dynamics of actin and myosin pointed to an effect of oxidation on weak-to-strong structural transition and by using site-directed mutagenesis of *Dictyostelium* (*Dicty*) myosin II oxidation, a redistribution of existing structural states of the actin-binding cleft was implicated (Prochniewicz et al., 2008b; Klein et al., 2011). Alterations in myosin heavy chain expression in uremic animals have also been reported (Taes et al., 2004).

Many studies have observed increased levels of oxidative stress biomarkers in blood samples of CKD patients (Samouilidou and Grapsa, 2003; Filiopoulos et al., 2009). In the literature, there are sufficient studies with different technical approaches in which the activity and role of ROS and reactive nitrogen species (RNS) in skeletal muscle has been studied using both *in vivo* and *in vitro* methods in a variety of contexts (Powers et al., 2011). Thus, based on recent advances in our understanding of how ROS and RNS affect muscle function, this mini-review aimed to examine if oxidative stress can contribute to muscle dysfunction in ESRD.

## Methods

A systematic review was conducted searching PubMed and Scopus by using the Cochrane and PRISMA guidelines. A comprehensive literature search was conducted from September 2014 until November 2014. We used PubMed, ScienceDirect and Scopus or Google Scholar to search for studies that investigated the relationship among (i) oxidative stress and uremic myopathy in humans, and (ii) markers of oxidative stress in the skeletal muscle of uremic patients on HD. Eligibility of the studies based on titles, abstracts and full-text articles was determined by two reviewers. Studies were selected using inclusion and exclusion criteria. We included only those studies that met the following criteria: they assessed oxidative stress markers in the skeletal muscle of patients on HD; they used human biopsies; they addressed randomized control trials, controlled trials, or clinical trials designed to evaluate oxidative stress in skeletal muscle in uremic patients on HD therapy; they were written in English.

## Results and discussion

Only three studies have examined the oxidative damage in human skeletal muscle of uremic patients on HD (Table 1). Their findings are discussed with reference to renal human blood findings and/or animal muscle findings either models of CKD or models of other conditions.

**Table 1:** **Summary results of the studies meeting the criteria of the present systematic review**.

**References**	**Total glutathione nmol/mg protein**	**GSS Gnmol/mg protein**	**SOD U/mg protein**	**MDA nmol/mg protein**	**CAT U/mg protein**	**PC nmol/mg of protein**	**Thiols nmol/mg protein**
**MARKERS OF OXIDATIVE STRESS IN MUSCLE TISSUE OF UREMIC PATIENTS**							
Lim et al., 2002a	–	–	–	0.065 ± 0.009↑	–	3.78 ± −0.14↑	–
Lim et al., 2002b	–	–	–	23.76 ± 6.06↑	–	24.9 ± 4.00↑	–
Crowe et al., 2007	≈24↑	≈2.6	≈20	≈0.28↓	≈11↓	–	≈79
**MARKERS OF OXIDATIVE STRESS IN MUSCLE TISSUE OF HEALTHY CONTROLS**							
Lim et al., 2002a	–	–	–	0.043 ± 0.005	–	2.97 ± −0.28	–
Lim et al., 2002b	–	–	–	7.67 ± 0.95	–	3.78 ± 0.14	–
Crowe et al., 2007	≈5	≈3.3	≈27	≈0.52	≈34	–	≈60

*GSH, glutathione; GSSG, oxidized glutathione; SOD, superoxide dismutase; MDA, malondialdehyde; CAT, catalase; PC, protein carbonyl; Thiols, protein thiol content. Arrows indicate statistically significant differences reported by authors*.

Lim et al. (2002a) found increased malondialdehyde (MDA) and protein carbonyls (PC) levels reflecting extensive oxidative damage to total protein content and lipids, in muscle suggested by the authors to be due to increased levels of inflammatory cytokines and to increased protein degradation. Increased levels of lipid peroxidation in blood samples of CKD patients during HD treatment has also been found elsewhere (Varan et al., 2010) and could enhance the susceptibility of LDL oxidation which is a major contributor in the genesis of atherosclerosis. The above observations, together with animal findings in the role of carbonyl stress in vascular injury (Chen et al., 2013) concur to a role of protein oxidation in long-term vascular damage which could impact overall vessel functionality and thus striated muscle's bioenergetics and function.

The same group also reported increased mitochondrial protein and lipid oxidative damage in skeletal muscle of uremic patients compared to age-matched controls (Lim et al., 2002b). The authors also reported mitochondrial DNA mutations, and overall oxidative damage to total cellular DNA, supporting a notion of attenuating regenerative and bioenergetics capacities of the skeletal muscles of renal patients. Also, mitochondrial DNA deletions have been observed in the skeletal muscle of ESRD patients similar with these found in the skeletal muscle of elderly subjects due to oxidative damage which probably contribute to the impaired mitochondrial energy metabolism that characterizes uremic patients (Lim et al., 2000).

It is considered that mitochondrial membranes are more likely to develop oxidative damage due to the relatively high amounts of lipid containing polyunsaturated fatty acids that they possess (Laganiere and Yu, 1993) and this would explain their increased oxidative damage. Given that mitochondria are considered the predominant source of ROS in muscle fibers (Davies et al., 1982; McArdle et al., 2001; Jackson, 2009), due to the elevated oxygen consumption that occurs with increased mitochondrial activity, especially during exercise (Powers et al., 2011) it is conceivable that damage to the mitochondria membrane might further compound their function as a ROS source causing more leaking. Moreover, it has been suggested that mitochondrial ROS leaking depends on fiber type both at resting basal respiration and at an increased respiration (as in exercise). In an animal saponin-treated muscle study of mitochondrial respiration, type IIb skeletal muscle fibers showed significantly higher free radical leaking compared to type IIa and I fibers at basal respiration (Anderson and Neufer, 2006).

If indeed the ROS load in the late stages renal skeletal muscle is high, that might in part explain the higher susceptibility of type II fibers to atrophy observed in end stage patients, where not only generalized muscle atrophy was observed but more prominent atrophy was seen in type II (especially IIx) vs type I fibers either in non-locomotory (Sakkas et al., 2003a) or locomotory muscle samples. Furthermore, on the possible role of the mitochondrial dysfunction in renal muscle atrophy it should be noted that de-innervation studies show that denervated muscle mitochondria release fatty acid hydro peroxides, mediated by calcium dependent phospholipase A_2_ (Bhattacharya et al., 2009). Such observations together with observations of an increased sensitivity of human aged mitochondria to apoptosis (Gouspillou et al., 2014), and the findings of Lim et al. (2002b) reviewed above, can collectively substantiate an important role for mitochondrial dysfunction, in the pathogenesis of uremic myopathy.

In contrast to the findings of Lim et al. and Crowe et al. (2007), reported decreased MDA content, increased total glutathione and no change in protein thiols content, superoxide dismutase (SOD), oxidized glutathione (GSSG) and catalase activity and concluded that there is no evident connection between oxidative stress and muscle atrophy in uremia. However, if one considers the age difference in the subjects of the conflicting studies one cannot discount the possibility that the much younger subjects, which also had spent less time on dialysis, of the Crowe et al. study had better preserved mitochondrial status. Adaptation of various antioxidant mechanisms can also explain some of the above differences. It should also be noted that in transgenic mice studies, the model overexpressing phospholipid hydroperoxide glutathione peroxidase, Gpx4-Tg (which is associated with mitochondrial and other membranes) protected against denervation atrophy and not the manganese superoxide dismutase (Sod2-Tg) or copper-zinc superoxide dismutase (Sod1-Tg) models, suggesting that the release of fatty acid hydroperoxides from mitochondria may be a more important factor in denervation-induced atrophy than superoxide and hydrogen peroxide (Bhattacharya et al., 2009).

**Figure 1:**
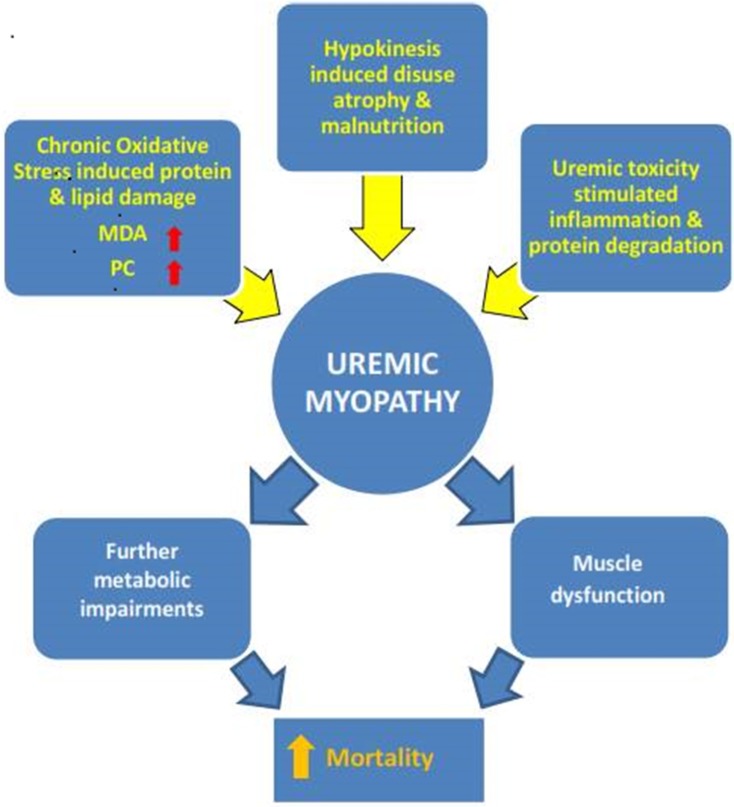
**The multifactorial nature of uremic myopathy**. Many specific disease-related but also lifestyle factors (e.g., physical inactivity) contribute to the pathological muscle state. Exactly when one factor reaches critical importance cannot be surmised so far. The results of this systematic mini review do point to oxidative stress as a contributor to the development of uremic myopathy. MDA, malondialdehyde; PC, protein carbonyls.

The above discourse further highlights the difficulty faced by researchers in renal patient studies. Many confounding factors such as years in dialysis, nutrition, physical activity levels, level of treatment, and comorbidities can affect muscle status and accelerate or decelerate disease and aging effects (Figure 1).

## Conclusions

CKD has a high and increasing prevalence not only in the old retirees but also in the middle-aged Europeans (Zoccali et al., 2010). Skeletal muscle dysfunction is a ubiquitous finding in CKD patients on advanced stages of the disease. The impact is grave as the statistics are implacable. Muscle loss and weakness contribute to the high morbidity and mortality of these patients, especially at the end-stage renal failure. Many specific disease-related but also lifestyle factors (such as physical inactivity) can be seen as contributors to the pathological muscle state. Exactly when one factor reaches critical importance cannot be surmised so far. The few studies meeting our search criteria while not agreeing, do point to a possibly important role for oxidative stress in uremic myopathy. It is not known if hypothesized oxidative stress mediated effects on muscle function are more of an acute or a chronic nature. *In vitro* studies however show clearly that oxidative stress does have a role whether via chronic, protein and other modifications or acute contractility effects. If anything the three studies and the peripheral literature highlight the need for a systematic study of the disease mechanisms affecting skeletal muscle performance in renal disease.

As long as great unknowns remain on the mechanisms and modulation of uremic myopathy, which leads to debilitation and premature death, progress in the management of this new epidemic is the least slowed down. We suggest that more muscle research, human and animal, should be done on pro-dialysis stages including work on the role of oxidative stress. This would allow researchers to decipher early changes, and perhaps identify susceptible individuals for accelerated muscle loss, before moving into the end-stage situation which on its own has detrimental effects on muscle status.

### Conflict of interest statement

The authors declare that the research was conducted in the absence of any commercial or financial relationships that could be construed as a potential conflict of interest.
